# Mass Spectrometry Imaging Reveals Spatial Metabolic Alterations and Salidroside’s Effects in Diabetic Encephalopathy

**DOI:** 10.3390/metabo14120670

**Published:** 2024-12-02

**Authors:** Shuohan Cheng, Xianyue Meng, Zixuan Wang, Tianfang Lan, Zhi Zhou, Zhonghua Wang, Zeper Abliz

**Affiliations:** 1 Key Laboratory of Mass Spectrometry Imaging and Metabolomics, Minzu University of China, National Ethnic Affairs Commission, Beijing 100081, China; shuohan_cheng@163.com (S.C.); massi6893@163.com (X.M.); wangzixuan1874@163.com (Z.W.); 23302551@muc.edu.cn (T.L.); zhouzhi@muc.edu.cn (Z.Z.); zeper@muc.edu.cn (Z.A.); 2 Key Laboratory of Ethnomedicine of Ministry of Education, School of Pharmacy, Minzu University of China, Beijing 100081, China; 3 Center for Imaging and Systems Biology, College of Life and Environmental Sciences, Minzu University of China, 27 Zhongguancun South Avenue, Beijing 100081, China

**Keywords:** mass spectrometry imaging, diabetic encephalopathy, AFADESI-MSI, salidroside, metabolic alterations

## Abstract

**Background:** Diabetic encephalopathy (DE) is a neurological complication of diabetes marked by cognitive decline and complex metabolic disturbances. Salidroside (SAL), a natural compound with antioxidant and neuroprotective properties, has shown promise in alleviating diabetic complications. Exploring the spatial metabolic reprogramming in DE and elucidating SAL’s metabolic effects are critical for deepening our understanding of its pathogenesis and developing effective therapeutic strategies. **Methods:** Air-flow-assisted desorption electrospray ionization–mass spectrometry imaging (AFADESI-MSI) was employed to investigate spatial metabolic alterations in the brains of db/db mice, a spontaneous DE model. The mice were treated with SAL (30 and 150 mg/kg, orally) for 12 weeks. Differential metabolites were identified and characterized using high-resolution mass spectrometry and validated against public databases. **Results:** Our AFADESI-MSI analysis revealed significant changes in 26 metabolites in the brains of DE mice compared to the controls. These metabolic changes indicated disruptions in glucose, glutamate-glutamine, nucleotide, lipid, choline, aspartate, and L-carnitine metabolism. Notably, glucose 6-phosphate (G6P), glutamine, adenosine, L-carnitine, and choline exhibited similar trends in both db/db mice and STZ-induced rat models of DE, suggesting their potential as reliable biomarkers. Twelve weeks of SAL treatment demonstrated a positive regulatory effect on glucose metabolism, the glutamate–glutamine cycle, and lipid metabolism. **Conclusions:** This study identifies key metabolic alterations in DE and demonstrates the therapeutic potential of SAL in modulating these disturbances, offering valuable insights for targeted interventions in diabetic complications.

## 1. Introduction

Diabetic encephalopathy (DE) is a neurological complication affecting a substantial portion of the diabetic population, with approximately 40% experiencing cognitive impairment [1,2]. Despite the increasing prevalence of diabetes, the underlying molecular mechanisms driving DE remain elusive, hindering the development of effective treatments [1]. Addressing the significant public health and economic burden of DE necessitates a comprehensive understanding of its pathophysiology to inform novel prevention and therapeutic strategies.

Metabolites are the end products or intermediate products of cellular processes in the brain. They play a critical role in brain function, influencing various aspects like energy production, neurotransmission, and cellular signaling [3,4,5]. The concentration of metabolites in different regions of the brain is closely linked to both normal and pathological processes. For instance, the concentrations of glucose and adenosine triphosphate (ATP) vary among different brain regions and reflect their respective energy demands [6]. Changes in the levels of neurotransmitters, such as glutamate and γ-aminobutyric acid (GABA), can significantly affect neuronal communication and cognitive function [7]. Factors such as age, diet, exercise, and disease can influence the distribution of metabolites in the brain, leading to changes in brain function [8,9,10,11]. In neurodegenerative diseases like Alzheimer’s, Parkinson’s, and DE, alterations in metabolite distribution are indicative of impaired energy metabolism and increased oxidative stress, which can result in cognitive decline and neuronal damage [12,13,14]. Thus, uncovering and locating metabolic biomarkers within the brain is crucial for both comprehending the underlying mechanisms of brain diseases and deciphering how drugs produce their therapeutic outcomes at the molecular level.

Mass spectrometry imaging (MSI) has emerged as a powerful tool for spatially resolving metabolite distributions within biological tissues, offering invaluable insights into the metabolic underpinnings of neurological diseases [15,16]. Techniques such as desorption electrospray ionization–MSI (DESI-MSI) and matrix-assisted laser desorption/ionization–MSI (MALDI-MSI) have unveiled distinct lipid profiles and metabolic pathways, respectively, in Alzheimer’s disease (AD) brains, highlighting their potential for biomarker discovery and therapeutic target identification [17,18].

Our group previously developed air-flow-assisted desorption electrospray ionization–MSI (AFADESI-MSI) to investigate metabolic alterations in a rat model of DE [11,12,13,14,15,16,17,18,19]. While this model has provided valuable insights, it is important to acknowledge the limitations of a single animal model in fully recapitulating the complexity of human DE.

**Figure 1:**
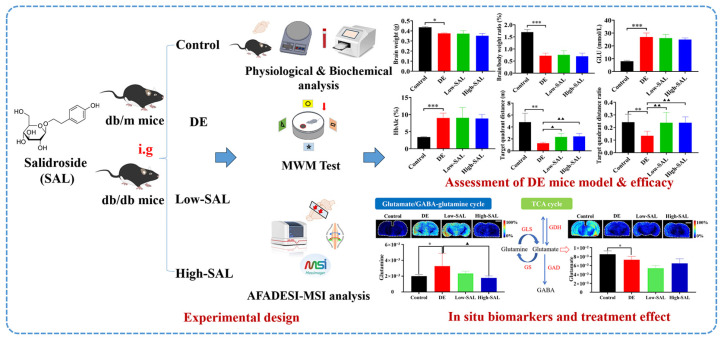
The research strategy for unveiling the spatial metabolic alterations and regulatory effects of SAL in DE using ambient mass spectrometry imaging. Comparisons with the normal control group are indicated as * *p* < 0.05, ** *p* < 0.01, and *** *p* < 0.01, and comparisons with the DE group are indicated as ^▲^ *p* < 0.05 and ^▲▲^ *p* < 0.01.

In this study, we employed AFADESI-MSI in a spontaneous DE model of 16-week-old db/db mice. By comparing these mice to healthy controls, we aimed to identify key metabolites associated with critical pathways in DE pathogenesis, revealing metabolic heterogeneity and reprogramming. Additionally, we evaluated the therapeutic potential of salidroside (SAL) in modulating these metabolic disturbances. The research workflow is summarized in Figure 1.

## 2. Materials and Methods

### 2.1. Chemicals and Reagents

**Figure 2:**
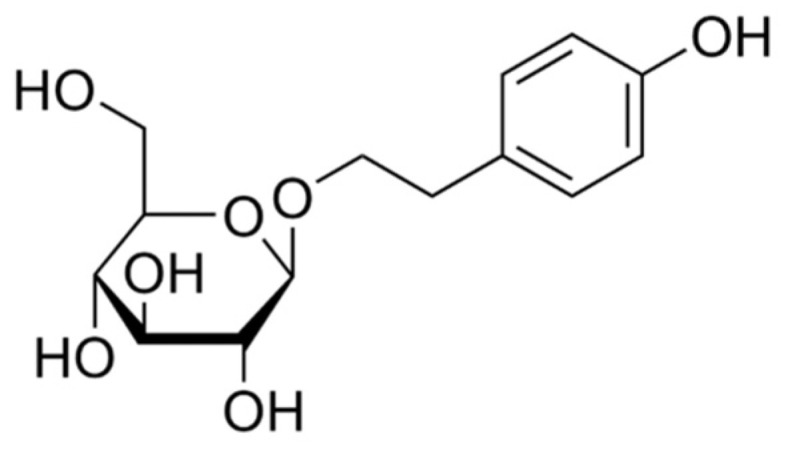
The structural formula of SAL.

HPLC-grade acetonitrile (ACN) and methanol (MeOH) were purchased from Merck (Muskegon, MI, USA). Purified water was obtained from Wahaha (Hangzhou, China). SAL was purchased from Chengdu Desite Biotechnology Co., Ltd. (Chengdu, China), with a purity of ≥98%. The compound’s partition coefficient (log *p*) was determined to be −0.39. It was extracted from the dried roots and rhizome of *Rhodiola crenulata* (Hook.f.et. Thoms.) H.Ohba. The structural formula is shown in Figure 2. It has been reported that the plasma concentration of SAL reaches a maximum (Cmax) at 0.5 h (Tmax) following intragastric administration in mice [20].

### 2.2. Animal Models

Four-week-old male BKS.DB mice (6 db/db homozygotes and 6 db/m littermate controls) were obtained from Gempharmatech Co., Ltd. (Nanjing, Jiangsu, China). Mice were housed under a 12 h light/dark cycle at a constant temperature of 23 ± 3 °C. The db/m littermates served as the control group, while the db/db homozygotes were randomly divided into three groups: DE (n = 6), Low-SAL (n = 6), and High-SAL (n = 6). The SAL was dissolved with a 0.9% sodium chloride injection. The Low-SAL and High-SAL groups received SAL intragastrically at doses of 30 and 150 mg/kg, respectively, once daily for 12 weeks. The control and model control groups were administered a 0.9% sodium chloride injection (vehicle) at a dose of 10 mL/kg. After 12 weeks, Morris water maze tests were conducted to assess spatial learning and memory. Blood and brain samples were collected and stored at −80 °C for subsequent analysis. All animal experiments were approved by the animal welfare ethics committee of Beijing Union-Genius Pharmaceutical Technology Development Co., Ltd. (Beijing, China), and adhered to the Guide for the Care and Use of Laboratory Animals.

### 2.3. Biochemical Analysis

Glycosylated hemoglobin (HbA1c) was measured using a Quo-Test HbA1c Analyzer (QUOTIENT Diagnostics Ltd., Walton-on-Thames, Surrey, UK). Blood glucose levels were determined using an AU480 automatic chemistry analyzer (Beckman Coulter Inc., Brea, CA, USA).

### 2.4. MWM Test

Spatial learning and memory were assessed using a circular water maze (120 cm diameter, 50 cm height) filled with water to a depth of 30 cm at a temperature of 24 ± 2 °C. A 15 cm diameter escape platform was submerged 2 cm below the water surface in the center of the southeast quadrant. A tracking system recorded escape latency and swimming paths. The mice were acclimatized for 30 min before testing. The water maze was connected to a computer for data acquisition. The mice were randomly assigned to one of four starting positions (east, west, south, or north). The time required to find the platform was recorded, followed by a 10 s rest period on the platform. The mice underwent daily training sessions for 2–5 days. On the day following the final training session, the platform was removed, and a 90 s probe trial was conducted. Mice were placed in the water opposite the original platform quadrant. The total distance traveled and the distance traveled in the target quadrant were recorded as indicators of spatial memory.

### 2.5. AFADESI-MSI Analysis

Frozen brains were embedded in saline medium, sectioned at 8 μm thickness using a cryostat, and thaw-mounted onto microscope slides. After vacuum-drying, the sections were analyzed using an AFADESI-MSI system equipped with a lab-made AFADESI ion source and a Q-OT-qIT hybrid mass spectrometer (Orbitrap Fusion Lumos; Thermo Fisher Scientific Inc., San Jose, CA, USA). Positive and negative MS spectra were acquired from 100 to 1000 Da with a spray voltage of ±7.0 kV, capillary temperature of 350 °C, spray gas pressure of 0.5 MPa, and transporting gas flow of 45 L/min. ACN/H_2_O (8:2, *v*/*v*) was used as the AFADESI spray solvent at a flow rate of 7 μL/min. The sprayer-to-tissue distance was 0.6 mm, the tube-to-tissue distance was 3 mm, and the orifice-to-tube distance was approximately 10 mm. During AFADESI-MSI experiments, brain tissue sections were scanned at a rate of 200 μm/s in the *x*-direction with a 200 μm step size in the *y*-direction.

### 2.6. Data Processing and Analysis

To process and analyze AFADESI-MSI data, raw files were converted to a .cdf format, imported into MassImager 2.0 for image reconstruction, and preprocessed by subtracting the background and delineating regions of interest. Average ion intensities within these regions were calculated and exported as .txt matrices. The data were then analyzed in Markerview 1.2.1 for background subtraction, peak extraction, alignment, and normalization to total ion current. *t*-tests and one-way ANOVA were used to compare differences between two or more groups. Results are presented as mean ± SD, with statistically significant differences defined as *p*-values < 0.05.

## 3. Results

### 3.1. Biochemical Analysis and MWM Test

**Figure 3:**
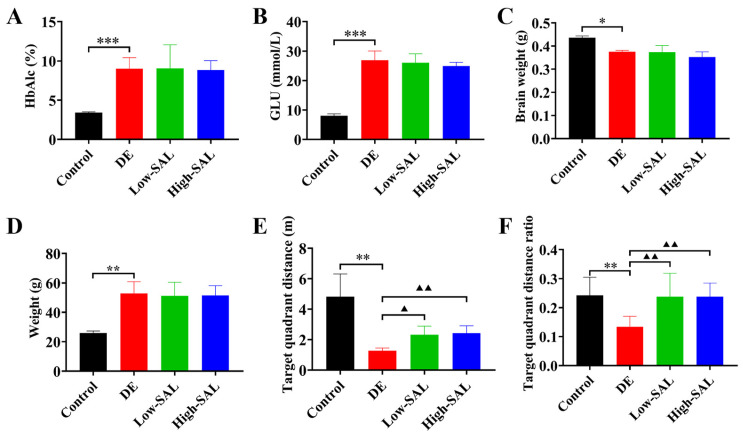
Physiological and biochemical changes in the Control, DE, Low-SAL, and High-SAL groups after 12 weeks. (**A**) Glycosylated hemoglobin; (**B**) Blood glucose levels; (**C**) Brain weight; (**D**) Body weight; (**E**) Target quadrant distance traveled in the water maze; and (**F**) Target quadrant distance ratio in the water maze. Comparisons with the normal control group are indicated as * *p* < 0.05, ** *p* < 0.01, and *** *p* < 0.01, and comparisons with the DE group are indicated as ^▲^ *p* < 0.05 and ^▲▲^ *p* < 0.01.

As depicted in Figure 3, the db/db mice exhibited hallmark features of DE, including hyperglycemia, elevated glycosylated hemoglobin, and brain atrophy. Consistent with these physiological abnormalities, these mice also demonstrated significant cognitive impairment in the Morris water maze. Notably, SAL treatment significantly ameliorated cognitive deficits in these animals, suggesting its potential therapeutic efficacy in mitigating the neurological complications of diabetes. While SAL did not directly influence blood glucose levels or other physiological parameters, these findings highlight its potential to intervene in the pathogenesis of DE.

### 3.2. AFADESI-MSI Analysis of Brain of db/db DE Mice

**Figure 4:**
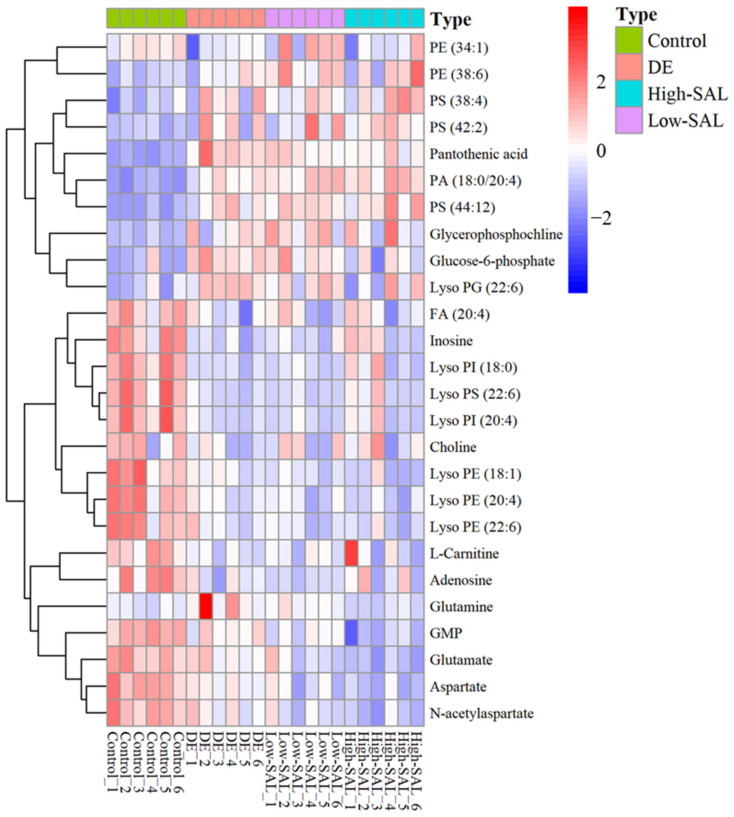
Heatmap displaying the differences in metabolite concentrations among the Control, DE, Low-SAL, and High-SAL groups.

To investigate the metabolic differences between DE and control mice, AFADESI-MSI data were analyzed using *t*-tests to identify statistically significant differential metabolites. These metabolites were further characterized with high-resolution mass spectrometry and compared to an in-house metabolite library and public databases such as HMDB and METLIN. In the positive ion mode of AFADESI-MSI analysis, 36 differential ions were detected, resulting in the identification of 5 metabolites. In the negative ion mode, 92 differential ions were identified, leading to the characterization of 21 metabolites. Among the 26 identified metabolites were 1 sugar, 4 amino acids, 3 nucleotides, 15 lipids, 2 cholines, and 1 carnitine (Table 1). After 12 weeks of treatment with a high dose of SAL (200 mg/kg), disturbances in these metabolites were ameliorated, particularly for glucose 6-phosphate (G6P) and glutamine (Figure 4).

### 3.3. Pathway Enrichment Analysis

**Figure 5:**
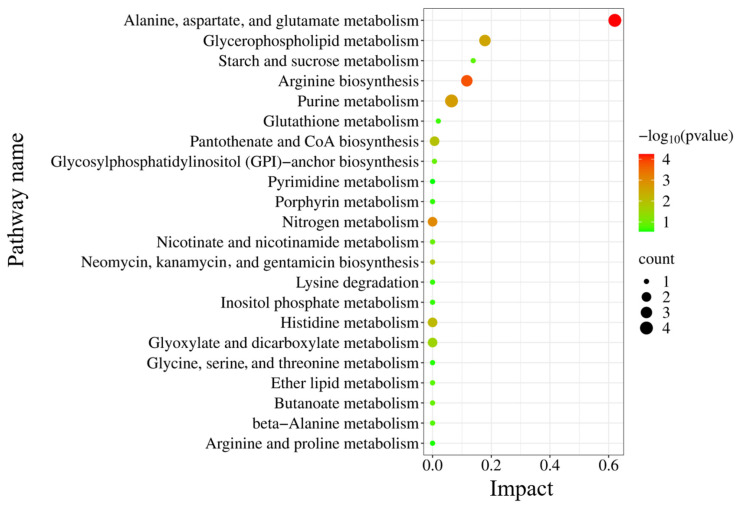
Pathway enrichment analysis of discriminating metabolites associated with DE in db/db mice.

The pathway enrichment analysis of discriminating metabolites was performed using MetaboAnalyst 6.0 (https://www.metaboanalyst.ca) (accessed on 1 September 2024) to identify metabolic pathways involved in db/db mice. As shown in Figure 5, a total of 22 metabolic pathways are associated with db/db mice. There are 8 items having impact values greater than 0. The top five affected pathways were alanine, aspartate, and glutamate metabolism; glycerophospholipid metabolism; starch and sucrose metabolism; arginine biosynthesis; and purine metabolism.

## 4. Discussion

### 4.1. Glucose Metabolism Disorder

**Figure 6:**
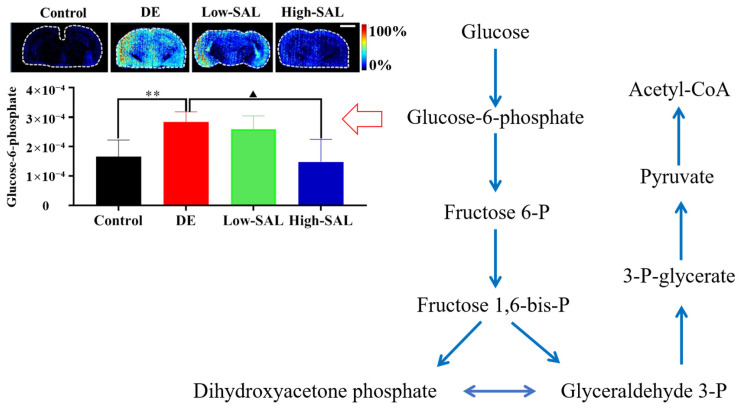
AFADESI-MSI screened the changes and distribution of differential metabolites related to glycolysis in the brains of mice in Control, DE, Low-SAL, and High-SAL groups. Comparisons with the normal control group are indicated as ** *p* < 0.01, and comparisons with the DE group are indicated as ^▲^ *p* < 0.05. Scale bar: 2 mm.

Glucose is a vital energy source for the brain, accounting for roughly 20% of total glucose intake despite the brain constituting only 2% of body weight. Proper regulation of glucose metabolism is therefore crucial for maintaining brain function [21]. In this study, we observed a significant increase in G6P levels in the brain tissues of DE mice compared to controls (*p* < 0.05), as shown in Figure 6. G6P is a key intermediate in glycolysis and the pentose phosphate pathway (PPP), both of which are essential for energy production and maintaining cellular redox balance [22]. The elevated G6P levels suggest impaired glycolysis, which could result in reduced ATP production, disrupting neuronal energy homeostasis and potentially contributing to the cognitive decline commonly observed in DE. Furthermore, higher G6P levels may drive the PPP, leading to an overproduction of NADPH and reactive oxygen species (ROS), thereby increasing oxidative stress [22]. This oxidative stress may promote neuronal damage and inflammation, factors involved in the progression of neurodegenerative diseases and diabetic complications. These findings are consistent with our previous observations in an STZ-induced rat model of DE [11].

After 12 weeks of treatment with SAL, the significant reduction in G6P levels, especially in the brains of the High-SAL group, suggests that SAL may effectively restore disrupted glucose metabolism in DE. The decrease in G6P levels indicates an improvement in glycolysis and a potential rebalancing of the PPP, which could enhance neuronal energy homeostasis. SAL’s ability to mitigate oxidative stress and support mitochondrial function has been well documented, providing a plausible mechanism for its role in regulating glucose metabolism in DE [23,24]. Furthermore, research has shown that SAL enhances insulin sensitivity by activating the AMPK pathway [25], which may explain its impact on reducing G6P levels and correcting metabolic dysfunction in DE.

### 4.2. Abnormalities in the Glutamate/GABA-Glutamine Cycle

**Figure 7:**
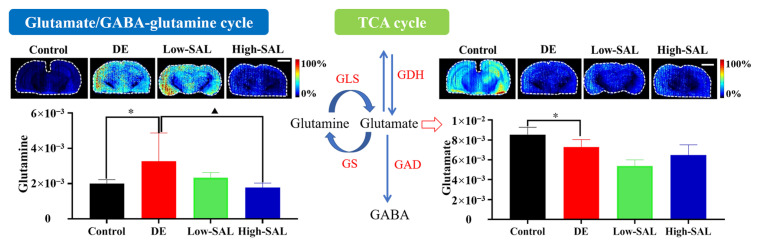
Spatial distribution and changes in the glutamate–glutamine cycle in DE. Comparisons with the normal control group are indicated as * *p* < 0.05, and comparisons with the DE group are indicated as ^▲^ *p* < 0.05. Scale bar: 2 mm.

The observed increase in glutamine levels and the corresponding decrease in glutamate levels in the brain of diabetic mice, as shown in Figure 7, suggest significant disruptions in the glutamate–glutamine cycle. This cycle is critical for maintaining neurotransmitter balance, as glutamate is the primary excitatory neurotransmitter in the brain, while glutamine serves as a precursor for both glutamate and GABA, the major inhibitory neurotransmitter [26,27]. Reduced glutamate levels could lead to insufficient excitatory neurotransmission, contributing to cognitive dysfunction and other neurological symptoms commonly observed in DE. Furthermore, excess glutamine accumulation may be linked to altered ammonia detoxification or disrupted astrocyte–neuron interactions, both of which are associated with neuroinflammation and oxidative stress, worsening neuronal health. The imbalance between glutamate and glutamine may also contribute to dysregulated energy metabolism and mitochondrial dysfunction in neurons, further exacerbating the cognitive decline characteristic of DE. These findings align with our previous observations in an STZ-induced rat model of DE, in which a significant increase in glutamine levels was detected, while glutamate levels showed no significant difference between the DE and control groups [11].

The observed trend of improvement in both high- and low-dose SAL administration groups, particularly the significant reduction in glutamine accumulation in the High-SAL group, suggests that SAL may play a regulatory role in restoring the balance of the glutamate–glutamine cycle in DE. SAL’s potential effect on the glutamate–glutamine cycle could be linked to its neuroprotective properties, including reducing oxidative stress and improving mitochondrial function. By enhancing mitochondrial efficiency, SAL might facilitate the proper conversion of glutamine to glutamate in neurons and astrocytes, helping to restore excitatory neurotransmission, which is disrupted in DE. Furthermore, the regulation of glutamine levels by SAL may reduce neuroinflammation and oxidative damage, both of which contribute to neuronal dysfunction in DE. Additionally, SAL may indirectly influence the activity of key enzymes such as glutamine synthetase and glutaminase, which are involved in the conversion processes of this cycle. By modulating these enzymes, SAL could help normalize the disrupted equilibrium between glutamate and glutamine, thereby improving cognitive function and mitigating the neurological symptoms associated with DE.

### 4.3. Disorder of Nucleotide Metabolism

**Figure 8:**
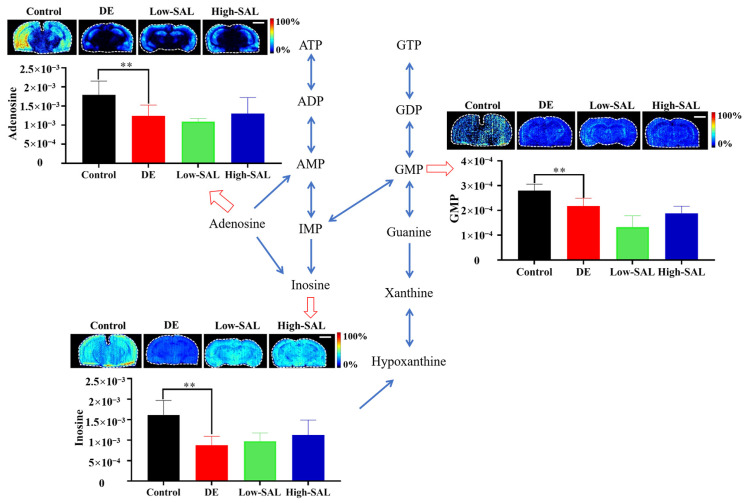
Spatial distribution and changes in the nucleotide-related metabolites in DE. Comparisons with the normal control group are indicated as ** *p* < 0.01. Scale bar: 2 mm.

A significant decrease in adenosine, inosine, and guanosine monophosphate (GMP) levels was observed in DE mice (Figure 8). Adenosine plays a dual role as an energy carrier (in the form of ATP) and as a neuromodulator in the central nervous system (CNS). A reduction in adenosine levels can impair synaptic transmission and reduce the brain’s ability to respond to energy demands [28], leading to cognitive deficits and increased neuronal vulnerability. Moreover, adenosine has anti-inflammatory and neuroprotective properties, and its depletion may exacerbate neuroinflammation and oxidative stress, both of which are implicated in DE pathology [29].

Inosine, a breakdown product of adenosine, has been shown to exert neuroprotective effects by promoting axonal regeneration and repair in damaged brain tissues [30]. The decrease in inosine levels may indicate reduced neuronal recovery capacity, thereby contributing to the progressive neurodegeneration seen in DE. Inosine also plays a role in purine metabolism, and its depletion may suggest overall disturbances in purine homeostasis, further affecting energy production and neuronal health.

GMP is involved in guanine nucleotide metabolism and serves as a precursor for the synthesis of guanosine triphosphate (GTP), which is essential for various cellular functions, including signal transduction and protein synthesis [31,32]. A decrease in GMP can impair the synthesis of GTP, affecting neurotransmitter release, synaptic plasticity, and neuronal signaling. This could contribute to the cognitive decline and neurological impairments observed in DE.

SAL treatment did not produce significant effects on these changes, indicating that while SAL may exhibit neuroprotective properties through other pathways, its influence on nucleotide metabolism, specifically the levels of adenosine, inosine, and GMP, remains limited in the context of DE.

### 4.4. Lipid Metabolism Abnormalities

**Figure 9:**
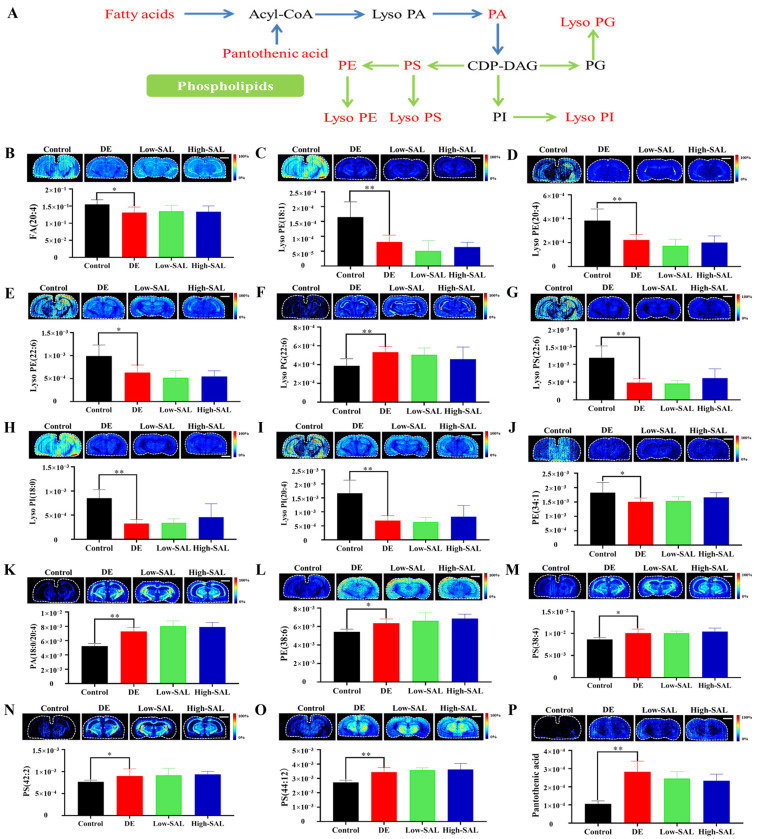
Spatial distribution and changes in lipid metabolism in DE. (**A**) Simplified pathway diagram of lipid metabolism; (**B**–**P**) air-flow-assisted desorption electrospray ionization–mass spectrometry images of metabolites involved in lipid metabolism. Comparisons with the normal control group are indicated as * *p* < 0.05, ** *p* < 0.01. Scale bar: 2 mm.

Lipids, essential components of biological membranes, play crucial roles in energy production, signaling, and maintaining membrane structure [33]. In lipid-rich tissues like the brain, disruptions in lipid metabolism are closely linked to disease progression [34]. This study identified significant alterations in lipid metabolism in DE. Several lipids, including FA (20:4), Lyso PE (18:1), Lyso PE (20:4), Lyso PE (22:6), Lyso PS (22:6), Lyso PI (18:0), Lyso PI (20:4), and PE (34:1), were significantly decreased in the brains of diabetic mice compared to controls. Conversely, pantothenic acid, Lyso PG (22:6), PA (18:0/20:4), PE (38:6), PS (38:4), PS (42:2), and PS (44:12) were significantly increased (Figure 9).

Notably, many of these differential lipids contain polyunsaturated fatty acids (PUFAs), indicating significant disruptions in PUFA homeostasis in the brain. PUFAs, such as arachidonic acid (FA 20:4) and docosahexaenoic acid (DHA, FA 20:6), are critical for maintaining membrane fluidity, modulating inflammation, and supporting cognitive function [35,36]. The observed alterations in PUFA-containing lipids suggest that the balance between their synthesis, utilization, and degradation is impaired in DE. This disruption may contribute to neuroinflammation, oxidative stress, and impaired neuronal signaling, all of which are central to the progression of diabetic complications in the brain. Decreased levels of PUFAs have been reported in various neurological diseases, including AD, Parkinson’s disease, and diabetic complications [37,38,39].

Compared to the diabetic group, high-dose SAL administration (150 mg/kg) tended to normalize the levels of FA (20:4), PE (34:1), Lyso PG (22:6), Lyso PS (22:6), Lyso PI (18:0), Lyso PI (20:4), and pantothenic acid, although not significantly. SAL notably improved the metabolic levels of certain PUFA-containing lipids, suggesting its potential to enhance learning and memory function and mitigate DE by modulating lipid metabolic pathways. Studies indicate that SAL may improve mitochondrial function, reduce oxidative stress, and restore PUFA balance [24,40,41], which are crucial for cognitive function and neuronal health in diabetic models.

### 4.5. Disordered in Choline, Aspartate, and L-Carnitine Metabolism

**Figure 10:**
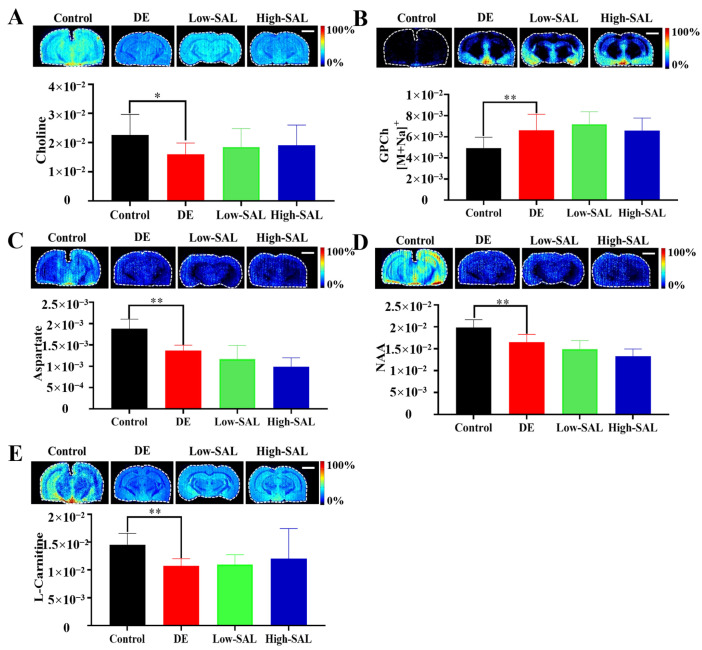
Spatial distribution and changes in choline, aspartate, and L-carnitine metabolism in DE. (**A**–**E**) air-flow-assisted desorption electrospray ionization-mass spectrometry images of metabolites involved in choline, aspartate, and L-carnitine metabolism. Comparisons with the normal control group are indicated as * *p* < 0.05, ** *p* < 0.01. Scale bar: 2 mm.

Choline levels were significantly reduced, while glycerophosphorylcholine (GPCh) levels were significantly elevated in the brains of the diabetic mice compared to the controls (Figure 10). Choline is a precursor for acetylcholine, a neurotransmitter essential for cognitive processes such as memory, learning, and attention [42]. It is also a component of phosphatidylcholine, a major phospholipid in cell membranes. Glycerophosphorylcholine is a breakdown product of phosphatidylcholine [43]. The observed alterations in choline and GPCh levels suggest potential disturbances in acetylcholine synthesis and membrane phospholipid composition, which may contribute to memory and learning impairments and impact overall neuronal health and connectivity. Changes in choline and GPCh levels have also been associated with AD and other neurological conditions [44].

Both aspartate and N-acetylaspartate (NAA) levels were significantly reduced in the brain tissue of diabetic mice compared to the normal group. Aspartate is an important amino acid that plays multiple roles in the brain. It is a key component of the excitatory neurotransmitter system and is involved in the synthesis of other neurotransmitters [45]. Aspartate participates in glutamate–aspartate neurotransmission, which is crucial for synaptic plasticity, learning, and memory [46]. NAA, a metabolite predominantly found in neurons, is often used as a marker of neuronal health and density [47]. NAA is involved in the synthesis of acetyl coenzyme A and contributes to neuronal energy metabolism [48]. The reduced levels of both aspartate and NAA in the brains of diabetic mice suggest significant disruptions in neurotransmission, neuronal metabolism, and overall brain function. These changes may contribute to the cognitive decline and neuropathological features observed in DE.

L-carnitine was significantly reduced in the brain tissue of the diabetic mice compared to the normal group. L-carnitine is essential for fatty acid metabolism, particularly within mitochondria, where it facilitates the transport of long-chain fatty acids for β-oxidation, a key energy-producing process [49]. A reduction in L-carnitine levels can impair brain cells’ ability to utilize fatty acids, leading to an increased reliance on glucose for energy [50]. This shift may worsen the metabolic abnormalities associated with diabetes. Moreover, L-carnitine possesses neuroprotective properties, including antioxidant and anti-inflammatory effects [51]. Lower levels of L-carnitine in the brain could further exacerbate oxidative stress and inflammation, contributing to the progression of neurological disorders [52].

Compared to the diabetic group, choline and L-carnitine levels showed a trend toward normalization with high-dose SAL administration, although the differences were not statistically significant. This suggests that SAL may have a potential modulatory effect on these metabolites.

## 5. Conclusions

This study employed AFADESI-MSI to investigate metabolic alterations in a spontaneous DE model of db/db mice. Our findings revealed significant disruptions in multiple metabolic pathways, including glucose metabolism, the glutamate–glutamine cycle, nucleotide metabolism, lipid metabolism, and the metabolism of choline, aspartate, and L-carnitine. These disturbances align closely with our previous observations in an STZ-induced rat model of DE and the findings of Dong et al. [11,12,13,14]. Specifically, G6P and glutamine were found to accumulate, while glutamate, choline, adenosine, inosine, aspartate, NAA, and L-carnitine were downregulated in these rodent models of DE. This underscores the common metabolic dysfunctions underlying different forms of DE and highlights key pathways for therapeutic intervention. We also identified metabolic alterations not reported in other studies, including a series of decreases in Lyso PE, Lyso PS, and Lyso PI, as well as increases in PA and PS. These imbalances in PUFA-containing lipids suggest that the db/db mouse model exhibits more pronounced disruptions in PUFA homeostasis compared to the STZ-induced rat model of DE.

SAL demonstrated therapeutic potential by modulating metabolic disturbances, particularly in glucose metabolism, the glutamate–glutamine cycle, and lipid homeostasis. Although the effects on nucleotide metabolism were limited, SAL’s neuroprotective, antioxidant, and mitochondrial-preserving properties suggest its potential as a therapeutic agent for DE. This study highlights the value of ambient mass spectrometry imaging in elucidating DE pathogenesis and underscores the promise of SAL as a therapeutic candidate.

## Figures and Tables

**Table 1:** Differential Metabolites Identified in DE by AFADESI-MSI.

Metabolite	Elemental Composition	Adduct	Theoretical *m*/*z*	Measured *m*/*z*	Delta (ppm)
Choline	C_5_H_13_NO	[M+H]^+^	104.1070	104.1069	−0.9
L-Carnitine	C_7_H_15_NO_3_	[M+H]^+^	162.1125	162.1123	−1.0
Glycerophosphocholine	C_8_H_20_NO_6_P	[M+H]^+^	258.1101	258.1093	−3.1
Adenosine	C_10_H_13_N_5_O_4_	[M+H]^+^	268.1040	268.1037	−1.2
Inosine	C_10_H_12_N_4_O_5_	[M+Na]^+^	291.0700	291.0698	−0.7
Aspartate	C_4_H_7_NO_4_	[M−H]^−^	132.0302	132.0299	−2.5
Glutamine	C_5_H_10_N_2_O_3_	[M−H]^−^	145.0619	145.0613	−3.9
Glutamate	C_5_H_9_NO_4_	[M−H]^−^	146.0459	146.0455	−2.6
N-acetylaspartate	C_6_H_9_NO_5_	[M−H]^−^	174.0408	174.0402	−3.4
Pantothenic acid	C_9_H_17_NO_5_	[M−H]^−^	218.1034	218.1031	−1.4
G6P	C_6_H_13_O_9_P	[M−H]^−^	259.0224	259.0219	−2.1
FA (20:4)	C_20_H_32_O_2_	[M−H]^−^	303.2330	303.2326	−1.2
GMP	C_10_H_14_N_5_O_8_P	[M−H]^−^	362.0507	362.0502	−1.4
Lyso PE (18:1)	C_23_H_46_NO_7_P	[M−H]^−^	478.2939	478.2938	−0.2
Lyso PE (20:4)	C_25_H_44_NO_7_P	[M−H]^−^	500.2783	500.2780	−0.5
Lyso PE (22:6)	C_27_H_44_NO_7_P	[M−H]^−^	524.2783	524.2774	−1.7
Lyso PG (22:6)	C_28_H_45_O_9_P	[M−H]^−^	555.2729	555.2724	−0.8
Lyso PS (22:6)	C_28_H_44_NO_9_P	[M−H]^−^	568.2681	568.2676	−0.9
Lyso PI (18:0)	C_27_H_53_O_12_P	[M−H]^−^	599.3202	599.3199	−0.5
Lyso PI (20:4)	C_29_H_49_O_12_P	[M−H]^−^	619.2889	619.2884	−0.8
PE (34:1)	C_39_H_76_NO_8_P	[M−H]^−^	716.5236	716.5237	0.2
PA (18:0/20:4)	C_41_H_73_O_8_P	[M−H]^−^	723.4970	723.4978	1.1
PE (38:6)	C_43_H_74_NO_8_P	[M−H]^−^	762.5079	762.5073	−0.8
PS (38:4)	C_44_H_78_NO_10_P	[M−H]^−^	810.5291	810.5283	−0.9
PS (42:2)	C_48_H_90_NO_10_P	[M−H]^−^	870.6230	870.6228	−0.2
PS (44:12)	C_50_H_74_NO_10_P	[M−H]^−^	878.4978	878.4973	−0.5

## Data Availability

The data are contained within the article.
